# DNA-based detection of *Mycobacterium avium* subsp. *paratuberculosis* in domestic and municipal water from Porto (Portugal), an area of high IBD prevalence

**DOI:** 10.3934/microbiol.2021011

**Published:** 2021-05-17

**Authors:** Telma Sousa, Marta Costa, Pedro Sarmento, Maria Conceição Manso, Cristina Abreu, Tim J. Bull, José Cabeda, Amélia Sarmento

**Affiliations:** 1 FP-ENAS (UFP Energy, Environment and Health Research Unit), Universidade Fernando Pessoa, Rua Carlos da Maia, 296 - 4200-150 Porto, Portugal; 2 Faculdade de Ciências da Saúde, Universidade Fernando Pessoa, Rua Carlos da Maia, 296 - 4200-150 Porto, Portugal; 3 Departamento de Biologia & CESAM, Universidade de Aveiro, Campus Universitário de Santiago, 3810-193 Aveiro, Portugal; 4 Institute of Infection and Immunity, St George's University of London, Cranmer Terrace London SW17 0RE, UK; 5 Escola Superior de Saúde Fernando Pessoa, Rua Delfim Maia, 334 – 4200-253 Porto, Portugal; 6 Centro Interdisciplinar de Investigação Marinha e Ambiental (CIIMAR/CIMAR), Avenida General Norton de Matos, 4450-208 Matosinhos, Portugal; 7 I3S - Instituto de Investigação e Inovação em Saúde, Universidade do Porto, Rua Alfredo Allen, 208 - 4200-135 Porto, Portugal

**Keywords:** *Mycobacterium avium* subsp. *paratuberculosis*, inflammatory bowel disease, MAP detection, water contamination

## Abstract

*Mycobacterium avium* subsp. *paratuberculosis* (MAP) may play a role in the pathology of human inflammatory bowel disease (IBD). Previously, we found a high frequency (98% in patients with active disease) of MAP DNA detection in the blood of Portuguese Crohn's Disease patients, suggesting this cohort has high exposure to MAP organisms. Water is an important route for MAP dissemination, in this study we therefore aimed to assess MAP contamination within water sources in Porto area (the residential area of our IBD study cohort).

Water and biofilms were collected in a wide variety of locations within the Porto area, including taps connected to domestic water sources and from municipal water distribution systems. Baseline samples were collected in early autumn plus further domestic water samples in early winter, to assess the effect of winter rainfall. DNA was extracted from all 131 samples and IS900-based nested PCR used to assess the frequency of MAP presence.

Our results show high MAP positivity in municipal water sources (20.7% of water samples and 41.4% of biofilm samples) and even higher amongst domestic sources (30.8% of water samples and 50% of biofilm samples). MAP positivity in biofilms correlated with positivity in water samples from the same sources. A significantly higher frequency of MAP-positivity was observed during winter rains as compared with samples collected in autumn prior to the winter rainfall period (61.9% versus 30.8%). We conclude that domestic and municipal water sources of Porto region have a high burden of MAP contamination and this prevalence increases with rainfall. We hypothesize that human exposure to MAP from local water supplies is commonplace and represents a major route for MAP transmission and challenge which, if positively linked to disease pathology, may contribute to the observed high prevalence of IBD in Porto district.

## 1. Introduction

*Mycobacterium avium* subsp. *paratuberculosis* (MAP), a member of the *Mycobacterium avium* complex, is the aetiological agent of Johne's Disease (JD), an enteritis particularly affecting ruminant species (bovine, ovine and caprine). MAP has a wide-ranging host spectrum, causing enteritis in many wild animal species, including deer, rabbits and macaques [1],[2]. Europe and North America consistently report moderate/high incidence of JD (resulting in important economic losses) that persist because of limited availability in efficient pathogen eradication policies that are disadvantaged mostly by the lack of a commercial vaccine able to fully prevent non-clinical faecal shedding [3]–[5].

Common pathological traits between JD and Crohn's disease (CD) in humans, along with consistent reports of MAP detection in CD patients [6],[7] have suggested a possible role of MAP in the pathology of this type of inflammatory bowel disease (IBD). However, MAP has not been detected in every CD patient and can be commonly found in the gut and peripheral blood of many apparently normal human controls [8]. The aetiology of IBD (including CD) is thus likely to be multifactorial including the presence of host genetic (and epigenetic) susceptibility traits [9],[10], composition changes in gut microbiota (dysbiosis) and long term colonization/ chronic exposure to particular pathobionts that include MAP and other species such as adherent-invasive *Escherichia coli* (EC) [7],[11]–[14]. These indications are supported from studies showing significant influencing factors in disease progression include diet and associated exposure to particular water and soil environments, many of which contain MAP and EC, and the long-term remission observed in a number of patients receiving either faecal transplantation and/or anti-MAP therapy [15]. The current evidence thus suggests that MAP may represent only one possible aetiological agent and that a variety of etiological factors contribute singular aspects to the triggering of this syndrome which in themselves exert diverse degrees of influence on individual hosts depending on existing susceptibilities.

Humans can become infected by MAP through consumption of meat or dairy products from contaminated animals [16] and the environment. Water is an important vehicle for MAP dissemination and source of human exposure [17]. Indeed, water is used in multiple ways (consumption, oral hygiene, vegetable growth) and is diet-independent, thus, not restricted to any one particular diet regimen. Inhalation of exposure from contaminated water aerosols may also be a route for MAP entry [18],[19]. Several studies have demonstrated that MAP remains viable for long periods (years) in soils and water, bound to solid particles in suspension and also inside amoebae [20]–[22].

A pharmaco-epidemiological study conducted between 2003–2007 and reported by Azevedo et al. [23] showed a trend to increasing inflammatory bowel disease (IBD) incidence in Portugal, in particular in the Lisbon and Porto areas. In previous work [24] we have shown high prevalence of MAP and EC DNA in the peripheral blood of Portuguese patients from the Porto area with CD and ulcerative colitis. We found a high frequency of CD patients positive for both MAP and EC DNA in blood, regardless of disease activity, suggesting that both microbial agents may play a part in CD progression. Interestingly, about 38% of the subjects included in our healthy control group were also positive for MAP DNA in blood, suggesting high exposure to MAP in the Porto area.

MAP has been shown to highly and persistently contaminate soils and water downstream of areas where animal farms were located [21],[25],[26]. In many of the suburban zones of Porto, cattle and dairy farming remains commonplace. High apparent MAP infection prevalence rates have been reported throughout Northern Portugal including cattle, sheep and wild animals such as boar and deer [27]. This suggests a consistent falloff of MAP from these sources into the abundant river systems from which all Porto potable water is reservoired and sourced. Thus, in the present work we aimed at assessing if MAP contaminated water is present in the human water supply chain of the Porto area, constituting a potential environmental risk for IBD that could help explain the high incidence we have previously found in this region.

## 2. Material and methods

### 2.1. Sample collection

**Figure 1.**
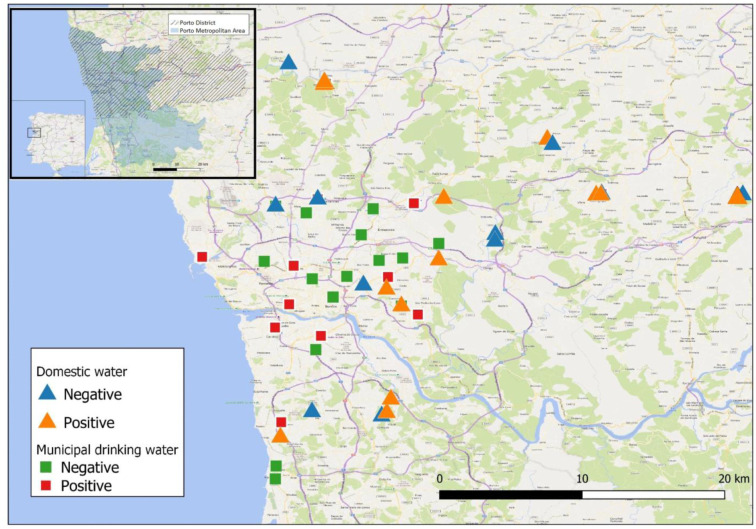
Geographical representation of Porto District/Porto Metropolitan Zone and distribution of domestic (triangles) and municipal (squares) collection sites. Red/orange symbols represent positive sites for MAP DNA and green/blue symbols represent negative sites for MAP DNA. MAP DNA detection was performed as described in Material and Methods. Dashed area, Porto District; shaded area, Porto Metropolitan Zone.

Water and biofilm samples were collected by volunteers from Universidade Fernando Pessoa in different municipalities of Porto Metropolitan Area (PMA) and/or Porto District (PD) (Figure 1). Samples were collected from taps connected to either domestic water sources (including household dug wells or pumped groundwater sources) or the municipal water distribution system. Porto municipal water is collected in the Crestuma-Lever catchment located in Douro river watershed, upstream the city of Porto, in the municipality of Vila Nova de Gaia.

Domestic sources comprised 26 collection sites from 9 municipalities (Amarante, Gondomar, Paços de Ferreira, Paredes, Maia, Trofa, Valongo, Vila do Conde and Vila Nova de Gaia). Municipal sources comprised 29 collection sites from 8 municipalities (Gondomar, Paredes, Porto, Maia, Matosinhos, Trofa, Valongo and Vila Nova de Gaia). Samples were collected initially in September/October 2018 (Collection 1) with additional 21 domestic water samples collected from 7 municipalities (Gondomar, Paços de Ferreira, Paredes, Maia, Trofa, Vila do Conde and Vila Nova de Gaia) in December/January 2019 (Collection 2) to evaluate possible effects of dilution caused by winter rains. Samples included also biofilm collections from taps accounting for a total of 131 samples of which 73 were domestic samples (26 water samples + 26 biofilm samples collected in autumn + 21 water samples collected in winter) and 58 municipal samples (29 water + 29 biofilm samples collected in autumn).

### 2.2. Water sample collection and filtration

Municipal (treated public water supply) and domestic (dug wells or groundwater) water samples (1 L) were collected using sterile water collection flasks (bacteriology grade) (VWR). Each sample was filtered under vacuum using a sterile filter funnel with a 0.45 µm membrane (MicroFunnel™, Pall Laboratories). Filter membranes were removed with sterile forceps, placed in a 5 mL PowerWater DNA bead tube (Qiagen) and frozen at −20 °C until processing for DNA extraction using the DNeasy PowerWater kit (Qiagen), according to manufacturer's instructions. At the end of the procedure, extracted DNA was eluted with solution EB (supplied with the kit), to a final volume of 100 µL.

### 2.3. Biofilm collection and preparation

Biofilms were collected in households from taps connected to the municipal drinking water system or to household water sources (dug wells or groundwater) [28]. Biofilm samples were collected using a cotton swab immediately immersed and shaken in 1 mL sterile water (molecular biology grade) (Sigma-Aldrich) to allow biofilm detachment. The swab was then discarded and the water containing biofilm residues centrifuged at 13,000 xg for 1 minute. Supernatant was removed and the pellet frozen at −20 °C until processed for DNA extraction using the DNeasy PowerBiofilm kit (Qiagen), according to manufacturer's instructions. At the end of the procedure, extracted DNA was eluted with solution EB (supplied with the kit), to a final volume of 100 µL.

### 2.4. MAP DNA detection by PCR

Confirmation of MAP DNA was carried out by real-time nested PCR (for increased sensitivity), using the real-time PCR LightCycler™ 1.5 Carousel-based system (Roche Applied Science). For the first round of PCR, primers L1 (CTTTCTTGAAGGGTGTTCGG) and L2 (ACGTGACCTCGCCTCCAT) were used [29]. These primers amplify a region in the insertion sequence IS900, resulting in a 398 bp amplification product. The PCR reaction was performed on LightCycler™ capillaries (20 µL) (Roche Applied Science) in a final volume of 20 µL, using the following reaction conditions: 10 µL SSO Advanced Universal inhibitor-tolerant SYBR Green Supermix, 4 µL of molecular biology grade water, 0.5 µL of each primer L1 and L2 (0.5 µM final concentration) and 5 µL of template DNA. Cycling conditions were as follows: Initial denaturation/enzyme activation at 95 °C for 3 min, followed by 25 amplification cycles consisting of 15 sec at 95 °C, 15 sec at 58 °C, 30 sec at 72 °C and fluorescence acquisition after 5 sec at 85 °C. Melting point analysis was then performed using 1 cycle at 95 °C for 15 sec, 47 °C for 15 sec and then continuous fluorescence monitoring while ramping to 96 °C at 0.2 °C/sec. Carrousel was then cooled to 30 °C for 30 sec. For the nested PCR, primers AV1 (ATGTGGTTGCTGTGTTGGATGG) and AV2 (CCGCCGCAATCAACTCCAG) were used [29]. The PCR reaction was performed in a final volume of 20 µL, using the following reaction conditions: 10 µL SSO Advanced Universal inhibitor-tolerant SYBR Green Supermix (Bio-Rad), 8 µL of molecular biology grade water, 0.5 µL of each primer AV1 and AV2 (0.125 µM final concentration) and 1 µL of template DNA. Cycling conditions were as follows: initial denaturation/enzyme activation at 95 °C for 3 min followed by 40 cycles of amplification consisting of 95 °C for 15 sec, 62 °C for 15 sec and 72 °C for 20 sec (fluorescence acquisition at end). Melting point analysis was then performed using 95 °C for 15 sec, 47 °C for 15 sec and then continuous fluorescence monitoring while ramping to 96 °C at 0.2 °C/sec. Carousel was then cooled to 30 °C for 30 sec. To minimize the risk for amplicon contamination, the PCR mixes were prepared in a clean isolated room and DNA samples used in the first and second round PCR were added in different laminar flow hood biosafety cabinets located in different rooms. As an additional safety measure these rooms were equipped with ceiling UV lamps. Both the biosafety cabinets and the rooms where they are located were UV-treated for at least 30 minutes before and after DNA addition. Reaction mixes were prepared in a separate room that was kept sample free at all times. Molecular biology grade water was used for the negative controls of both PCR rounds. This was added to the corresponding PCR capillary at the end of sample addition, keeping the capillary open during the entire process. Positive control DNA was prepared from a CFU quantified MAP culture aliquot. The positive control used was close to the sensitivity limit. Sensitivity of the assay was determined by 10 fold serial dilutions of CFU quantified MAP culture dilutions and found to be of 20 CFU per reaction (data not shown). Filter tips and molecular biology grade reagents and disposables were used throughout the procedure.

### 2.5. Statistical analysis

Comparison of MAP DNA prevalence between domestic and municipal water and/or biofilm samples, as well as between domestic water collection 1 and 2, was made using Fisher's exact test. In all tests, a significance of P < 0.05 was considered. The program Prism 9 (GraphPad Software, San Diego, CA, USA) was used for statistical analysis and graphical construction.

### 2.6. Design of geographical maps

Geographical maps were constructed using the QGIS 3.16 software for windows (https://qgis.org/en/site/).

## 3. Results and discussion

### 3.1. Geographical distribution of collection sites

Water/biofilm collection sites are depicted in Figure 1. Since water and biofilms from domestic sources were collected from taps connected to household dug wells or to pumped groundwater sources, collection sites were more likely to be located in peripheral municipalities of Porto metropolitan area or in the Porto district, in less urbanized areas. Contrastingly, the majority of municipal drinking water and biofilm samples were obtained more centrally and associated with more urbanized areas.

### 3.2. MAP contamination of domestic and municipal water sources

**Figure 2.**
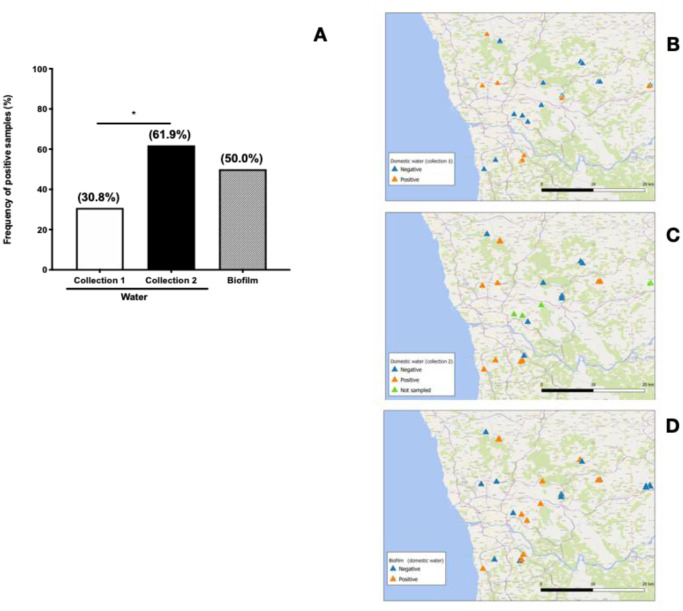
MAP contamination of domestic water sources. (A) Frequency of samples from domestic water sources positive for MAP DNA. Water samples were collected prior (Collection 1) and during (Collection 2) the winter rainfall period. Biofilm samples were collected at Collection 1 time point, only. MAP DNA was extracted and analysed as described in Materials and Methods. (B),(C),(D) Geographical distribution of domestic water samples positive and negative for MAP DNA. (B) Water sample results at Collection 1 time point, (C) Water sample results at Collection 2 time point and (D) biofilm sample results. *p < 0,050.

**Figure 3.**
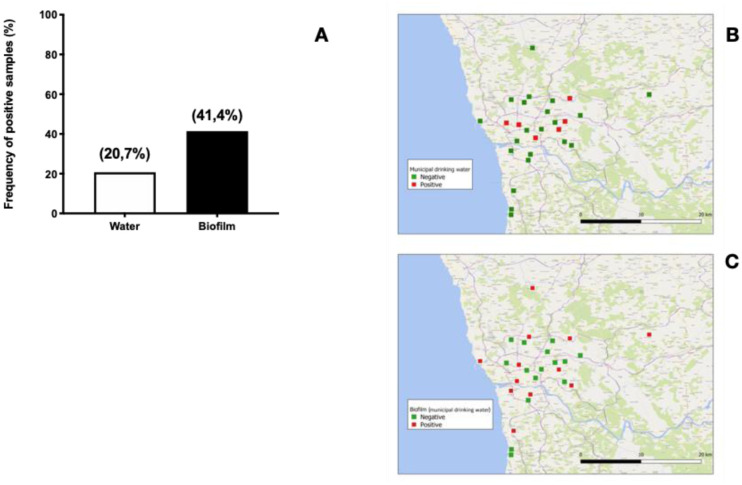
MAP contamination of municipal drinking water sources. (A) Frequency of samples from municipal water sources positive for MAP DNA. Water samples were collected at Collection 1 time point (prior to winter rains). MAP DNA was extracted and analysed as described in Material and Methods. (B) and (C), Geographical distribution of positive and negative samples for MAP DNA in municipal water sources. (B) water sample results, (C) biofilm sample results.

In this study, MAP DNA was more frequently detected in domestic samples (domestic water 8/26 or 30.8%; municipal water 6/29 or 20.7%; domestic biofilm 13/26 or 50.0%; municipal biofilm 12/29 or 41.4%). Although differences between domestic and municipal water sources were statistically not significant (Figures 2 and 3) our results are in line with the findings reported by Pickup et al. [21].

It has been reported that soil and rivers near cattle farms show heavy MAP contamination [16],[21],[25],[26], which leads to contamination of groundwater that is sourced for domestic water collection. Water treatment can substantially reduce MAP contamination, as described in a number of studies where MAP was more frequently detected upstream than downstream of water treatment works [21],[30]. This might be explained mostly by removal of suspended organic solids, since MAP associates with these particles but is resistant to chlorination [31]. Nonetheless, water treatment processing does not totally eliminate MAP organisms and some may escape to form biofilms on water pipes, building up over time and leading to persistent contamination of drinking water systems [20],[32]. Indeed, we found a higher trend for MAP positivity in biofilm samples collected from both domestic and municipal sources as compared to their corresponding water samples (Figures 2 and 3), although these differences were not statistically significant.

The frequency of treated municipal water samples positive for MAP DNA in our survey (6/29 or 20.7%) was considerably higher than previously reported in South Wales (2%) [21], Italy (3%) [33] and a national US survey (0%) [28], but lower than reported in Northern Ireland (45%, by IS900 PCR) [30] and a US midwest region (80%) [28]. These differences are not attributable to the type of detection as all these studies used PCR. However, at least partially methodological issues may still play an important role as in some cases small samples (50 mL) were used [21] and this may severely impact the sensitivity of the detection [21]. In one of the cited studies, culture assays, were also used in parallel but found to render negative results even in cases where as high as 50% of samples turn PCR positive [30], a finding that may be explained by the difficulty of culturing MAP. Together these findings led us to choose to test MAP presence exclusively by the Nested qPCR and to use 1 L sample volume for the present study to minimize false negatives. Our findings suggest that both domestic water and municipal drinking water may be an important source of MAP contamination and transmission in the Porto region.

### 3.3. Effects of rainfall on MAP contamination

A significantly higher frequency of MAP-positivity was observed among domestic water samples collected during winter rains (Collection 2–13/21 or 61.9%), as compared to samples collected before winter rains, at the beginning of autumn (Collection 1–8/26 or 30.8%) (Figure 2). Increased river height and flow rate was associated with higher MAP detection on rivers Taff and Tywi, at South Wales, UK [21],[25]. Higher MAP contamination was also found on river Tywi when heavy rainfall was observed in the preceding days of sample collection [21]. In a comprehensive geographical survey on soil samples conducted in Great Britain, Rhodes and collaborators found a strong association between MAP contamination and the presence of cattle farms [26], although soil contamination was widespread and not confined to cattle areas. They also found that MAP contamination was decreased in soils located in areas of higher rainfall, suggesting that soil runoff can be a major contribution for a rise in river and aquifer MAP contamination. Although not included in the present study, effects of winter rains in the catchment water would also be important to evaluate, since a rise in MAP contamination during winter can also impact municipal treated water. Future work can address this hypothesis.

Publicly available data on existing animal farms (2019 agricultural census from the Portuguese National Statistical Institute–INE) does not have sufficient geographical resolution to allow comparison with our data. Nevertheless, the north of Portugal (including the Porto region) was an area of high cattle breeding until the late 1970's, for milk or meat consumption, with some cattle and sheep farms still remaining nowadays, including in the peripheral municipalities of Porto area as reported for the broad agricultural Northern Region of Portugal in the 2019 agricultural census from the Portuguese National Statistical Institute–INE. Although information on paratuberculosis prevalence in northern Portugal is limited, a serological study using ELISA for detection of anti-MAP antibodies on 3900 sheep belonging to 150 flocks in northeast Portugal showed that 46.7% of flocks had positive animals. According to the ELISA test sensitivity and specificity, the true paratuberculosis prevalence was 6.7% [34]. In another study, MAP DNA (IS900) was detected in faeces from 22 out of 24 asymptomatic bovines belonging to 4 farms in diverse locations of northern Portugal [35]. Positive MAP culture was also obtained from 12 faecal samples among the 22 MAP DNA positive ones [35]. These reports point to high paratuberculosis prevalence in animal farms of northern Portugal, which may lead to soil contamination by animal shedding and subsequently to water contamination by soil runoff.

## 4. Conclusions

We conclude that domestic and municipal water sources of Porto region show evidence of MAP contamination, making it a potential environmental IBD risk to take into consideration. Differences in the presence of MAP before and after rainfall suggests that it can be associated with soil runoff to rivers and groundwater from animal sources leading to the formation of MAP biofilms on water pipes. We hypothesize that this represents a major route for human exposure and colonization of MAP which, if able to trigger disease states in susceptible hosts, could contribute to the observed high prevalence of IBD in Porto district.
